# Transcriptomic Crosstalk between Gliomas and Telencephalic Neural Stem and Progenitor Cells for Defining Heterogeneity and Targeted Signaling Pathways

**DOI:** 10.3390/ijms222413211

**Published:** 2021-12-08

**Authors:** Roxana Deleanu, Laura Cristina Ceafalan, Anica Dricu

**Affiliations:** 1 Institute for Neuroscience, Medical University of Innsbruck, 6020 Innsbruck, Austria; Irina-Roxana.Deleanu@i-med.ac.at; 2 Department of Cell Biology and Histology, Carol Davila University of Medicine and Pharmacy, 050474 Bucharest, Romania; lauraceafalan@yahoo.com; 3 Victor Babeș National Institute of Pathology, 050096 Bucharest, Romania; 4 Department of Biochmistry, University of Medicine and Pharmacy, 710204 Craiova, Romania

**Keywords:** single-cell RNA-seq, primary cerebral tumors, glioma, cancer stem-like cells, human neural stem cells, human neural progenitors, neurogenesis, gliogenesis

## Abstract

Recent studies have begun to reveal surprising levels of cell diversity in the human brain, both in adults and during development. Distinctive cellular phenotypes point to complex molecular profiles, cellular hierarchies and signaling pathways in neural stem cells, progenitor cells, neuronal and glial cells. Several recent reports have suggested that neural stem and progenitor cell types found in the developing and adult brain share several properties and phenotypes with cells from brain primary tumors, such as gliomas. This transcriptomic crosstalk may help us to better understand the cell hierarchies and signaling pathways in both gliomas and the normal brain, and, by clarifying the phenotypes of cells at the origin of the tumor, to therapeutically address their most relevant signaling pathways.

## 1. Introduction

Deciphering the composition of the human brain and its primary tumors continues to be one of the central concerns in neuroscience and neuro-oncology. Over recent years, several international large-scale efforts have been devoted to analyzing and understanding normal and diseased brain composition, development and functions—including its oncological aspects. However, the classification and characterization of the cells of the brain and brain tumors is very challenging, and no consensus has yet been achieved.

The adult human brain is composed of two main cell compartments: a larger differentiated cell compartment which contains several hundred billion neuronal, glial and non-neural cells [1,2], and a much smaller compartment of potentially proliferative cells—including neural stem and progenitor cells—responsible for the modest cell turnover of the adult brain. However, new neurons and glia are continuously produced from various types of stem and progenitor cells throughout life in restricted brain areas. Unfortunately, some of these cells can suffer genetic alterations, producing different types of primary brain tumors—mainly gliomas—with an incidence of around 5 new cases per 100,000 people every year [3].

Malignant gliomas, of which glioblastomas are the most common [3], are associated with different genomic alterations and are some of the most lethal cancers. Several glioma subtypes relate to mutations in the enzyme isocitrate dehydrogenase (IDH). Mutations in *IDH1*, or less frequently *IDH2*, define two major classes of malignant gliomas: astrocytoma (IDH-A) and oligodendroglioma (IDH-O). Their distinct morphology and oligodendroglial or astrocytic marker expression suggest different glial lineages. However, a mixture of cells with histological features of neoplastic astrocytic and oligodendroglial cells are frequently observed within individual tumors, making the existence of distinct glial lineages in different IDH-mutated gliomas questionable [4].

IDH wild-type (IDHwt) glioblastoma is the most prevalent form of adult primary brain cancer. Analysis of whole-tumor transcriptomic data has shown that IDHwt glioblastoma includes three main subtypes: proneural (MGH26); classical (MGH30); and mesenchymal (MGH28, MGH29) [4,5]. However, all IDHwt glioblastoma individual tumors are highly heterogeneous, each containing different percentages of neoplastic cell types also present in all the other subtypes [4].

Moreover, all these malignant tumors are composed of two cellular compartments: a larger differentiated cell compartment, and a smaller compartment of cells with stem and progenitor features generically named “cancer stem-like cells” (CSCs), which means that they can self-renew and differentiate into multiple cell types, continually contributing to the tumor maintenance. CSCs isolated from different glioma, named glioma stem-like cells (GSCs), show variability with respect to marker expression, proliferation and differentiation, pointing to interpatient and intratumoral heterogeneity within the proliferative cell compartment as well [6]. GSCs were first identified through isolation of CD133-positive cells from primary glioblastoma, demonstrating that these cells were necessary and sufficient to give rise to an ectopic tumor [7]. A number of other neural stem cell markers, such as SOX2 and nestin, were used to validate the stemness of the GSC populations [6]. However, the current understanding of the stem and progenitor like-cell phenotypes within tumors and of their contribution to tumor heterogeneity and maintenance is still limited [8]. Although the GSC compartment is small in comparison to the differentiated compartment, it is still clinically relevant. Several studies have shown that GSCs resist radiotherapy and chemotherapy and are the main contributors to cancer recurrence. Presently, there are no clinically approved treatments specifically targeting GSCs—this is considered the main cause of the poor response to treatment in malignant gliomas [3,8].

In order to solve this problem, several recent studies have aimed to clarify the heterogeneity of GSCs and how their phenotypes link to the neural stem cells or the glial progenitor cells in the developing and adult brain. As most of the malignant glioma localize at the cerebral level, and many in the vicinity of the adult neural stem cell niches [4], the neural stem and progenitor cells in the developing and adult human telencephalon are the most relevant populations to be addressed. The updated knowledge about the neural development stages, cell hierarchies and phenotypes in humans is presented briefly, with the aim of better understanding the links they have with the neural stem, progenitor and differentiated cells in adult telencephalon, but also to address the hypothesis that different progenitors are specifically affected in different types of gliomas. 

Recent single-cell high-throughput approaches have allowed the taking of huge steps ahead in the definition of cellular identities and have provided unprecedented details on cellular diversity. The deep sequences of single cells or nuclei combined with bioinformatics tools provide the scale for an unbiased survey of molecular expression [9]. These tools can now overcome some of the previous difficulties associated with the scarcity of human brain tissue and can be applied to relatively small neurosurgical or postmortem samples [10,11]. Several reported transcriptomic profiles coming from different human telencephalon stages and regions have increased our current understanding of the developmental dynamics of the three major neural cell types in the human brain: neurons, astrocytes and oligodendrocytes. 

At the same time, many databases nowadays include single cell sequencing of primary tumor specimens from human brains. These molecular profiles have captured the great extent of intra-tumor heterogeneity and have identified different GSC populations in different classically defined gliomas. Some recent studies, which are further briefly overviewed, have reported that several proliferating cells inside adult glioma samples show a remarkable similarity to different neural stem and progenitor cells found during normal telencephalic development. This supports the connection between neural development, neural stem cell niches and cancer biology, requiring a deeper consideration and opening new perspectives in cancer therapy.

## 2. Neural Cell Types in the Human Developing Telencephalon

The development of the nervous system in general implies a precise temporal and spatial generation for each cell type, following the stages of neural induction, patterning/proliferation, neurogenesis, gliogenesis and functional maturation [12,13,14,15,16,17,18]. 

**Figure 1:**
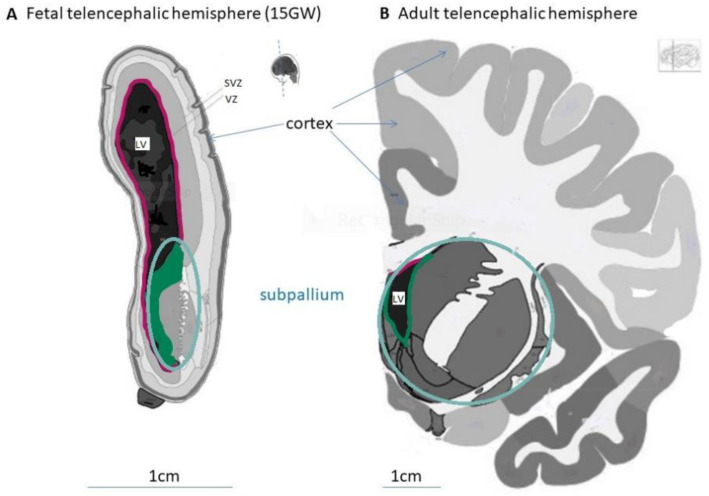
Morphological structures of the fetal and adult human telencephalon. Coronal sections from fetal (**A**) and adult (**B**) telencephalic hemispheres showing the pallium with the cortex, the subpallium and the lateral ventricle (LV). Images modified from the BrainSpan Reference Atlases for 15 gestational week and 34-year-old human brains sectioned at the rostral level (https://atlas.brain-map.org/, accessed on 20 August 2021).

*Neural induction* starts in the early human embryo at the middle of gestation week (GW) 3 in the midline anterior ectoderm, which transforms into the neuroectoderm. The neuroectoderm first organizes as a neural plate, which further extends and forms neural folds; they gradually fuse to form the neural tube, which is entirely closed at the end of the GW 4. In parallel with the neural tube forming and closing, the cells in the neuroectoderm transform. The initial neuroepithelial (NE) cells express the transcription factors (TF) PAX6 and SOX2, intermediate filaments such as nestin (NES), and adherent junction proteins such as N-cadherin (NCAD), zonula occludens 1 (ZO1) and prominin 1 (PROM1 or CD133) [19,20,21]. NE cells begin a transition into more elongated, radial-oriented cells called radial glia (RG), with their somas located in the ventricular zone (VZ) of the neural tube wall, their apical processes in contact with the internal surface (lumen or ventricle), and their basal processes contacting the external surface (pia matter) of the neural tube. In addition, by *pattering*, these RG acquire different identities in the anterior–posterior (A–P) and dorsal–ventral (D–V) axes due to gradients of morphogens produced by different organizer centers. A–P patterning starts in the head region by defining the forebrain (prosencephalon) and continues with the midbrain and the hindbrain. The forebrain further divides into the telencephalon and diencephalon. Parallel D–V patterning in the telencephalon leads to the definition of two main regions: the dorsal telencephalon or pallium and the ventral telencephalon or subpallium (Figure 1). 

Concomitantly, RG situated at different positions in the neural tube *proliferate* at different rates in response to local mitogens [16,22], forming morphologically defined domains and subdomains (Figure 1A). Several subtypes of RG with distinct behavioral, morphological, and transcriptional signatures have recently been described in prenatal human development [23,24]. Some RG, called ventricular radial glia (vRG, also called apical RG), remain situated in the VZ and continue to express NE cell markers, while others gradually move their somas into the newly formed subventricular zone (SVZ). After GW 9, many of them lose contact with the lumen of the neural tube and become the outer radial glia (oRG), locating and proliferating into the outer SVZ (oSVZ) and specifically expressing *HOPX* and the cell surface marker PTPRZ1, while losing the expression of some vRG markers [24]. By GW 34, some vRG detach basally and form truncated radial glia (tRG), which will be present during all of the following fetal stages [23]. The features of RG division are complex and tightly controlled in time and space. Symmetrical or asymmetrical mitosis occurs in response to different signals, parts of the FGF-MAPK cascade—including platelet-derived growth factor (PDGF) signaling [25]—the PI3K/PTEN/AKT pathway [26], hedgehog signaling [27], N-cadherin/β-catenin signaling [28], and Notch signaling [29].

*Neurogenesis*, the developmental process by which the neurons are generated, includes the production of neuronal progenitor cells and their differentiation into mature neurons. Between GW 5 and 28, RG dominate in all VZ, SVZ and oSVZ regions and produce most of the neurons in the human telencephalon, mainly indirectly, by generating transit-amplifying progenitors such as the intermediate (or basal) progenitor cells (IPCs) [30]. By asymmetric division, both vRG and oRG generate IPCs, which may undergo few symmetric divisions in response to different local mitogens. IPCs further differentiate into neurons, which use the RG processes as scaffolds to migrate superficially toward the basal (or marginal) zone of the neural tube [31]. The location of the neurons links tightly to the timing of their generation. This temporal patterning results in the sequential generation of specific types of neurons and is a fundamental process of neuronal diversification [18,32]. Generally, long projection neurons (excitatory and inhibitory) are produced first, followed by interneuron production. Excitatory neurons (EN), including glutamatergic neurons, are produced from pallial domains, while inhibitory neurons (IN), including GABAergic and cholinergic neurons, are produced from subpallial domains [16]. The neurons born in the subpallium can remain in ventral regions or migrate to the dorsal regions, as is the case with cortical inhibitory neurons [14,22,33]. During cerebral cortex formation, the first neurons migrating from the dorsal SVZ into the telencephalon mature into cortico-thalamic neurons. The most recently born neurons migrate past the earlier-born neurons and mature into intra-telencephalic neurons [18,34], while the migrating interneurons reach the cortex with a specific temporal and spatial distribution [22].

*Gliogenesis*, the developmental process by which glial cells are generated, includes the production of glial progenitor cells and their differentiation into mature glia. In the brain, these are macroglia (astrocytes, ependymal cells and oligodendrocytes) and microglia. Gliogenesis in the human telencephalon mainly occurs after the completion of neurogenesis, when the remaining RG in the VZ and SVZ proceed to produce macroglia directly or indirectly, while microglial cells have mesodermal origins [35]. The mode of transition from RG to mature astrocytes, ependymal cells and oligodendrocytes is still controversial and several models are proposed.

Most of the studies on astrocytogenesis have relied on the detection of intermediate filament proteins such as glial fibrillary acidic protein (GFAP), which are expressed by mature astrocytes but also by fetal and postnatal RG [36], making the interpretation of their expression difficult. Different types of RG could be direct or indirect sources of protoplasmic astrocytes in the gray matter and fibrous astrocytes in the white matter, as well as of the ependymal cells lining the ventricular system, which in the telencephalon form the lateral ventricles (LV; Figure 1). By GW 34, tRG appear to transform mainly into astrocytes, both fibrous and protoplasmic, that populate widespread regions of the brain parenchyma and express mature astrocytic markers such as AQP4 and APOE [35]. In the postnatal period, a few astrocytes can also undergo symmetric division and generate daughter astrocytes—a process that can also be detected in adult life [35]. Immature ependymal cells also arise from RG in parallel with astrocytes, and, by GW 34, they start to differentiate into mature ependymal cells, responsible for producing cerebrospinal fluid. From postnatal day 10 to adulthood, all ependymal cells lining the LV acquire a mature multi-ciliated morphology [37].

Oligodendrocytes are the myelin-forming glia in the central nervous system. As shown by several lineage-tracing studies in animal models, oligodendrocyte origins are both spatially and temporary diverse [38,39,40]. Multiple progenitor domains generate oligodendrocyte progenitor cells (OPCs) at distinct embryonic stages in the developing mouse telencephalon and several similar phenotypes have been identified in the human embryonic and fetal brain. Many studies have identified a variety of molecular markers for OPCs: platelet-derived growth factor receptor α (PDGFRα), chondroitin sulfate proteoglycan 4 (CSPG4) (also known as NG2), basic helix-loop-helix TFs OLIG1 and OLIG2, as well as the Sox family of high mobility group-containing TFs. OPCs begin to emerge as early as GW 5 and are continuously produced throughout the rest of the prenatal period, while migrating toward various brain regions, thereby becoming abundant and widespread already at birth [39]. Early born OPCs emerge from the most ventral progenitor domains and spread to more dorsal domains at later prenatal stages, with progenitors in the pallium becoming the major source of OPCs at early postnatal stages. OPCs derived from ventral progenitor domains mainly populate the subpallium, while those from both ventral and dorsal domains differentiate into oligodendrocytes which later myelinate the axons of the neurons in the dorsal telencephalon, such as the neocortex and corpus callosum. OPCs can stay undifferentiated for a long period of time during fetal and early postnatal stages, eventually becoming myelin-expressing mature oligodendrocytes at later stages [38,40]. Starting at early postnatal stages, OPCs begin to differentiate first into pre-myelinating immature oligodendrocytes (Pre-OL), defined by the expression of TFs NKX2.2 and NKX6.2, which subsequently undergo maturation into myelin-expressing oligodendrocytes [38]. While there are few oligodendrocytes and little myelination before birth, their number and the myelin produced by them in the white matter expand rapidly after birth until approximately 5 years of age [41].

The precise cell hierarchies and mechanisms that control the transition from RG to macroglia in different human telencephalic regions, as well as the ontogenic relationship between oligodendrocytes and astrocytes are still subjects of debate. The occurrence of transit-amplifying glial IPCs and migrating glioblasts, such as the neurogenic IPCs and migrating neuroblasts in neurogenesis, was also speculated for the astrocyte and oligodendrocyte lineages, but their exact identity and timing are currently unknown [35]. Cell culture experiments have demonstrated that OPC-like cells behave as bi-potent progenitors that can differentiate into both oligodendrocytes and astrocytes. Several in vivo studies, however, have demonstrated that OPCs become oligodendrocytes almost exclusively. Yet, some recent studies using genetic lineage-tracing methods have provided evidence that a fraction of PDGFRα/NG2-expressing cells differentiate not only into oligodendrocytes, but also into astrocytes and/or neurons in certain regions of the brain, although such cells seem to be relatively rare. A stepwise differentiation of RG via a bipotent glial progenitor cell (GPC), which may share markers and differentiate into both OPCs and astrocytes, has been proposed [42,43]. Recent data coming from single cell transcriptomics have started to clarify this issue (Section 4).

## 3. Neural Cell Types in the Human Adult Telencephalon

Differentiated neural cells such as neurons, oligodendrocytes, astrocytes and ependymal cells compose the large compartment of the adult telencephalon. Less than 1% of the neurons in the human brain, which are situated at pallial and subpallial levels in hippocampus and striatum, respectively, are replaced during adult life [44,45,46]. Unlike neurogenesis, adult gliogenesis remains active in the whole brain. Most OPCs generated during embryonic and early postnatal periods remain undifferentiated in the mature brain parenchyma throughout life, gradually becoming mature oligodendrocytes and replacing existing oligodendrocytes that are lost physiologically or after injury. The production of oligodendrocytes and myelin is very active postnatally and in early childhood—especially in the white matter—gradually decreasing toward the adult stages, when only 1 in 300 oligodendrocytes is replaced every year [41]. However, it is likely that postnatal gliogenesis is dynamically modulated in humans by learning new skills, with neuronal activity in general being associated with the generation of new oligodendrocytes and increased myelination in working brain regions. The production of new astrocytes in the adult brain parenchyma in normal conditions is even lower than the production of oligodendrocytes [47]. Somewhat unexpectedly, in certain pathological situations such as experimental stroke, astrocytes in the brain parenchyma can acquire an activated stem cell behavior, enter a neurogenic program, and give rise to new neurons, as well as new astrocytes. This supports the hypothesis of a widespread distribution of quiescent or “dormant” adult neural stem cells (NSCs) with an astrocytic-like phenotype [48,49,50].

However, it is recognized that new telencephalic neurons can normally be produced by adult NSCs, which are present only in two distinct niches: a pallial one situated in the subgranular zone (SGZ) of the hippocampal dentate gyrus [45] and a subpallial one in the SVZ lining the LV [51]. While hippocampal neurogenesis in the SGZ is mostly similar in mice and humans, the neurogenesis in SVZ is different. Large numbers of neurons generated from SVZ progenitors are continuously added to the olfactory bulb in adult rodents, but adult olfactory bulb neurogenesis could not be detected in adult humans [52]. Instead, human SVZ progenitors produce a subpopulation of interneurons detected in the striatum, which mainly express the marker calretinin [53]. However, adult neurogenesis at very low levels in other areas cannot be disregarded and continues to be an active area of exploration [49].

Adult NSCs in both the SVZ and SGZ derive from prenatal RG and share astrocyte morphology and markers, such as GFAP, but also stem cell markers such as CD133, SOX2 and nestin. The SVZ lining the LV in the human brain is composed of four distinct layers: a monolayer of ependymal cells alongside the ventricular cavity; a hypocellular space containing mainly GFAP-positive cellular processes; a dense layer containing mainly cells expressing GFAP (both adult NSCs and astrocytes), a smaller population of proliferating and migrating cells; and a transition zone adjacent to the parenchyma, mainly composed of oligodendrocytes and microglia. The proliferative marker KI67 (or the gene *MKI67*) is expressed in a limited number of cells in the SVZ, reflecting a very small number of cycling cells, which may co-express GFAP and SOX2 with OLIG2 or ASCL1, and the number of which decreases with age [37]. Although the adult NSCs, derived from various locations of the LV wall, can self-renew and behave as multipotent progenitors in both human and rodents—meaning that they produce all three neural lineages in vitro—whether each individual cell indeed produces both neurons and glia in vivo remains uncertain. Adult NSCs from the rodent SVZ, named B cells, undergo asymmetric cell divisions to give rise to a new B cell (one of the hallmarks of a stem cell), as well as to a transit-amplifying progenitor cell—also known as a type C cell. The progenitors further differentiate to only one distinct subset of neurons that migrate toward the olfactory bulb [54,55]. It is also currently unknown whether the same neurogenic adult NSCs can produce glial cell types in vivo. Again, a stepwise differentiation of the adult NSCs via intermediate precursor cells such as the bipotent GPCs has been proposed in rodents [42,43] and partially clarified by the scRNA-seq profiling of the adult SVZ in rodents and the transcriptomic crosstalk with human prenatal telencephalic progenitors (Section 4).

## 4. Single-Cell Transcriptomics of the Human Telencephalon

Transcriptomic sequencing is a technique that uses high-throughput, next-generation sequencing approaches to reveal the presence and quantity of RNA in a biological sample at a given moment. Recent advances in RNA-seq include single cell and single nucleus RNA-seq (scRNA-seq and snRNA-seq, respectively) [10,56,57]. Unlike bulk RNA sequencing, which interrogates average gene expression in cell populations that are in most cases heterogeneous [58], scRNA-seq can elucidate heterogeneity and allow cell-type specific transcriptomic profiling to be performed. The recent advent of high-throughput microfluidic systems with droplet-based profiling techniques has further advanced the precision of sc/sn RNA-seq profiling. Initially limited to only a few hundred cells per experiment, due to advances in experimental technologies, more than 1 million single cell transcriptomes can be profiled nowadays [59].

Several studies have performed single-cell sequencing of human embryonic fetal and adult samples of the human telencephalon [60,61,62,63,64,65,66,67,68,69]. In order to analyse the complex sets of single-cell data, robust computational methodologies need to be applied [56]. Unsupervised approaches use clustering followed by cluster annotation of cell types based on differentially expressed marker genes [70], while supervised approaches use a reference panel of labelled transcriptomes to guide both clustering and cell type identification [71]. Clustering of human telencephalic cell types has been obtained using unsupervised and supervised methods, or by using a combination of both. Each cluster was attributed with “unique markers”, which are the genes expressed only in that type of cell among all the cells sampled, as well as with “combinatorial markers”, which are differentially expressed genes that are not restricted to a single cell type. A consensus approach was proposed for both the clustering paradigms in order to increase the accuracy of the clustering and the precision of cell type annotation [72], which is expected to be applied in future studies.

**Figure 2:**
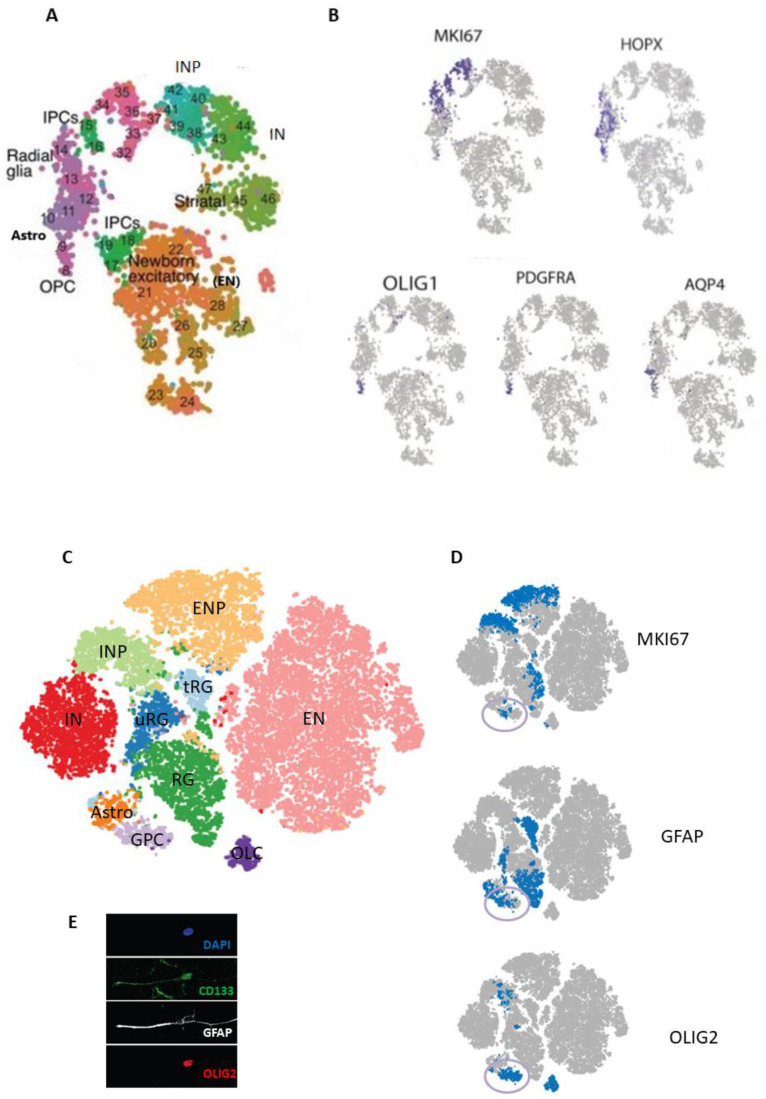
Single-cell RNA sequencing in the developing human telencephalon. (**A**). Plot of neural cells from pallial and subpallial human samples across prenatal stages (gestational weeks 6–37), colored by cluster and subcluster cell assignments, corresponding to different types of radial glia, intermediate progenitor cells (IPCs), excitatory neurons (ENs), inhibitory neuron progenitors (INPs), inhibitory neurons (INs), astrocytes (Astros), and oligodendrocyte progenitor cells (OPCs). (**B**). The same cluster representation as in (**A**), showing the cell/cluster-related expression of cycling and cell0specific genes (RG, OPCs and Astros). (**C**). Plot of neural cells isolated from the telencephalon of four human fetuses (gestation weeks 13–21), in which data sets from total neural cells and CD133+-selected cells were combined, colored by clusters representing three types of radial glia (marked as RG, truncated RG (tRG) and unknown RG (uRG), excitatory neuronal progenitors (ENPs), ENs, INPs, INs, Astros, glial progenitor cells (GPCs) and oligo-lineage cells (OLCs). (**D**). The same-cluster representation as in (**C**), showing the cell/cluster-related expression of the genes of the cycling and cell-specific genes (RG, OPCs and Astros); some cells in the GPC cluster co-express these markers. (**E**). Immunofluorescence image of human fetal telencephalic cells in primary culture co-expressing the proteins CD133, OLIG2, and GFAP. DAPI nuclear staining (blue). (**A**,**B**) adapted from [73], and (**C**–**E**) adapted from [74].

Nowakowski et al. performed scRNA-seq in human pallial and subpallial samples across prenatal stages from GW 6 to 37 [73]. Using unbiased clustering followed by a supervised approach using a reference panel of labelled transcriptomes, they identified transcriptionally distinct cell clusters and subclusters corresponding to RG, dorsal IPCs or excitatory neuron progenitors (ENP), excitatory neurons (EN), inhibitory neuron progenitors (INP), inhibitory neurons (IN), astrocytes (Astros), and OPCs (Figure 2A), as well as non-neural cell types: microglia, choroid plexus cells, mural cells, and endothelial cells. As expected, the proliferation gene *MKI67* was expressed in the known transit-amplifying populations of INPs and IPCs, as well as in a subpopulation of RG; *PDGFRA* and *OLIG1* were expressed mainly in the OPC cluster; the oRG gene *HOPX* was widely expressed in the RG cluster, and the mature astrocytic gene *AQP4* was restricted to the astrocyte cluster (Figure 2B). In addition, the lineage reconstruction method enabled the inference of gene expression trajectories from heterogeneous developmental tissue. Correlation of sample age with gene co-expression networks defined a maturation score, or “pseudoage”. Analysis of gene expression trajectories across “pseudoage” confirmed that early human cortical RG showed enriched expression of proneural transcription factors, whereas genes involved in gliogenesis are upregulated later in development. In addition, microdissected VZ and OSVZ samples were used to define a lamina score, or “pseudolamina”. Gene co-expression networks correlated with “pseudoage” and “pseudolamina” supported the classification of RG subtypes as vRG, oRG and tRG. These transcriptomic results indicate multiple signalling pathways which act during RG diversification and show the temporal and topographical hierarchy in dorsal and ventral telencephalic lineages of developing cortical neurons; within the dorsal telencephalon, these temporal and typological differences define progenitors across cortical areas, while topographical distinctions predominate across maturing neurons [73].

Couturier et al. performed scRNA-seq on freshly isolated cells from the telencephalon of four human fetuses ranging from GW 13 to 21 [74]. Fluorescence-activated cell sorting (FACS) was used to remove most of the non-neural cells such as microglia and endothelial cells, and CD133-positive selection improved the resolution of the neural stem and progenitor cell populations. Data sets of total and CD133-positive selected cells from all fetal brains were combined in silico after excluding ependymal cells and were used for unbiased grouping of cells into 10 clusters (Figure 2C). Differential gene expression analysis of these clusters identified important genes in each cluster. Most of these clusters correspond to previously defined cell populations in the developing human telencephalon [73], such as excitatory neuron progenitors (ENPs, corresponding to dorsal IPCs), ENs, INPs, INs, astrocytes (Astros) and oligodendrocyte lineage cells (OLCs, which may include OPCs and Pre-OLs), as well as three different RG clusters. However, the RG clusters were found to only partially correspond to the expression found in the pallial vRG, oRG, tRG or subpallial RG, and they were labelled as RG, tRG and uRG (undefined RG; Figure 2C). The proliferation gene *MKI67* was expressed, as expected, in the transit-amplifying neuronal progenitor clusters, but also in RG subpopulations—especially in the RG cluster—while *GFAP* was expressed in subpopulations in both the tRG and RG clusters. This suggests that the RG and uRG clusters correspond to mixed pallial and subpallial subpopulations of previously defined oRG and vRG, respectively. 

**Figure 3:**
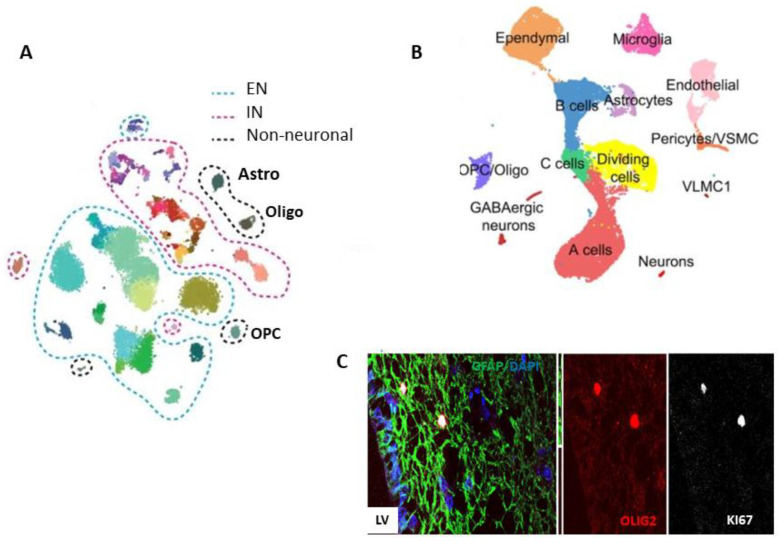
Single-cell RNA sequencing in the adult telencephalon. (**A**). Plot of cells from human adult cortex samples showing clusters and subclusters of neuronal populations (excitatory neurons—ENs and inhibitory neurons—INs), as well as small non-neuronal clusters of astrocytes (Astros), oligodendrocytes (Oligos) and oligodendrocyte progenitor cells (OPCs; adapted from Hodge et al., 2019). (**B**). Plot of cells from adult mouse subventricular zone (SVZ) samples (adapted from Dulken et al., 2017). (**C**). Immunofluorescence image of an adult human SVZ sample. Distribution of the cells lining the lateral ventricle (LV) and co-expressing KI67, OLIG2, and GFAP markers. DAPI nuclear staining (blue; adapted from [74]).

Interestingly, a glial progenitor cell (GPC) cluster was detected at all gestational ages and strongly expressed oligodendrocyte lineage genes (e.g., *OLIG1*, *OLIG2*, and *PDGFRA*), glial/astrocytic lineage genes (e.g., *GFAP*, *SOX9*, *HOPX*, *HEPACAM*, and *VIM*), and progenitor genes (e.g., *ASCL1*, *MKI67*, and *HES6*). However, it did not express several differentiation markers found in astrocytes or oligodendrocyte-lineage cell clusters (Figure 2D). This mixed gene signature partially differs from the signature of previously defined OPCs [73], but may be compatible with that of the proposed bidirectional GPC [42,43]. Notably, this GPC signature was almost exclusively identified in CD133-positive sorted cells, in a fairly small cluster, which likely explains why it was not previously detected in sequenced unsorted brain cell populations in both the prenatal and adult brain [60,61,62,63,64,65,66,67,68,69]. Importantly, the confirmation of cells co-expressing astro-like and OPC-like markers was done in a primary culture at first passage obtained from one of the cell-sequenced fetal brains (Figure 2E), as well as in the SVZ of the adult human brain (Figure 3C) [74]. This indicates a special subtype of progenitor population that is present from the fetal to the adult stages in the human brain. Additional bioinformatics helped to define a roadmap of the developmental-related trajectories in the human prenatal telencephalon, where these GPCs are linked to three differentiated neural lineages—represented here by interneurons, astrocytes and immature oligodendrocytes—while tRG link solely with the astrocyte lineage [74].

Velmeshev et al. performed snRNA-seq of human adult cortex samples and unbiased clustering, followed by annotation according to expression of known cell type markers, which identified 17 cell types, including subtypes of EN, IN and astrocytes [66]. Hodge et al. provided a more detailed transcriptomic map of the cells in the human adult cortex by following the same computational analysis as used in the mouse cortical cells previously profiled by Tasic et al. [64,75]. The transcriptional analysis of nuclei isolated from samples of human cortex revealed 69 neuronal and 6 non-neuronal clusters. From the neuronal clusters, 24 represented different types of EN, and 45 represented different types of IN. The major clusters of non-neuronal cells expressed SCL1A3 and included two astrocyte (Astro) types with different laminar distributions, OPCs, oligodendrocytes (Oligos) (Figure 3A), microglia, and endothelial cells. Astrocytes in the first cluster expressed higher *GFAP* and *AQP4* levels than the astrocytes of the second cluster; the first group may represent the interlaminar and fibrous types (from the connected white matter), while the second group represent the protoplasmic type. Cells in the OPC cluster expressed a high level of *PDGFRα*, which was also expressed at lower levels in the IN subpopulation, suggesting a common developmental-related pathway [64]. Addressing the composition and cell hierarchies of the known proliferative and neurogenic niches in the adult telencephalon, a single-cell RNA sequencing experiment investigated the rodent SVZ [76] (Figure 3B). Interestingly, the clusters representing the SVZ astrocytes, B cells and ependymal cells were closely related, while C, A, and the dividing cells form a quasi-continuum. OPCs formed a small separate cluster, but their hierarchic relationship with a subcluster of C cells, which may be the GPCs, should be further explored. To complement the studies in rodents, similar single-cell transcriptomic studies in human SVZ are expected to confirm these neurogenesis and gliogenesis pathways, but also to clarify the dilemma of the “dormant” NSCs in the adult human telencephalon [49].

## 5. Single-Cell Transcriptomics in Gliomas

Paralleling the extensive development of single cell transcriptomics of normal brain tissue, many databases and reports include single cell sequencing of brain tumor specimens. Several strategies have been used to exclude non-malignant cells, which are critical components of the brain tumor microenvironment, and to properly group malignant cells. FACS with negative selection for non-neural cells and computational filtering by using copy number variation (CNV) are the most used approaches to classify cells as belonging to malignant or normal tissues [74,77,78].

In addition, different known genetic alterations and expression of cell cycling, stemness and class-specific genes were investigated at the single cell level, and in some cases compared with their expression in normal human brain cells. The generated high-throughput molecular profiles captured to a great extent the intra-tumor heterogeneity and identified several populations of GSCs in different gliomas.

### 5.1. IDH Mutant Gliomas

With the aim of understanding the differences between the two major types of IDH-mutated diffuse gliomas—including the cells of origin—samples from oligodendroglioma (IDH-O) and astrocytoma (IDH-A) were first sequenced at the single-cell level by Tirosh et al. [79]. Each tumor included in the study contained a large population of cells with confirmed *IDH1* or *IDH2* mutations and co-deletion of chromosome 1p and 19q arms, as well as tumor-specific CNVs. Highly consistent across all IDH-O and IDH-A tumors, two prominent cell clusters expressed distinct lineage markers of oligodendrocytes and astrocytes, respectively. One cluster was strongly associated with the high expression of oligodendrocyte markers (such as *OLIG1/2*) and the low expression of astrocytic markers (such as *GFAP* and *APOE*), while the other cluster had the opposite expression patterns. A smaller cluster highly expressed genes related to neurodevelopment and neural stem cells, such as *SOX2*, *SOX4* and *ASCL1*. Remarkably, cells with OPC gene expression, which were suggested to represent the origin of oligodendrogliomas in a mouse model [80], did not form a separate cluster. All proliferating cells found in each tumor (1.5–8%), consistent with Ki67 expression, grouped in the small cluster. Together, this analysis revealed three main expression patterns recapitulating a stem/progenitor program of early neural development and subsequent differentiation into oligodendrocytes and astrocytes. The single-cell profiles suggested that the tumor-initiating cells in IDH-mutated gliomas more closely resembled the NSC type than a more committed glial progenitor type such as OPCs.

Following the study of Tirosh et al. [79], Venteicher et al. combined scRNA-seq results from ten IDH-A and six IDH-O tumor samples with bulk data from large cohorts from The Cancer Genome Atlas (TCGA) [81]. They found that differences in bulk expression profiles between IDH-A and IDH-O were explained primarily by genetic alterations and the composition of the tumor microenvironment (TME), but not by distinct glial expression programs in the malignant cells. Again, in both IDH-A and IDH-O tumors, only a small proportion of cells (~4% on average) were in a proliferative stage, co-expressing cycling and putative stem cell markers. Single-cell approaches showed again that undifferentiated cells from both tumor types exhibited increased similarity in gene expression programs, further suggesting a shared cell of origin for both IDH-A and IDH-O. Thus, IDH-mutant gliomas as defined by genetics and histopathology as differing in terms of genetics and TME but, examined at single-cell resolution, all contain three subpopulations of malignant cells: two non-cycling differentiated glial lineages—astrocyte-like and oligodendrocyte-like cells—as well as one cycling undifferentiated subpopulation that resembles NSCs.

Together, the studies on IDH mutant gliomas represent a shift in understanding the histogenesis of glial tumors and support a model where IDH mutant glioma subclasses share developmental programs and putative lineages of glial differentiation, but differ primarily by the genetic mutations and the number of macrophages and microglia in the TME [81].

### 5.2. Glioblastoma

IDHwt glioblastoma was the first brain tumor investigated at the a single-cell transcriptome level [78]. As for IDH mutant gliomas, it was shown clearly that bulk transcriptomics did not capture the true diversity of transcriptional subtypes within a tumor but detected only the dominant transcriptional program. While the classification of IDHwt glioblastoma via bulk transcriptomics includes the proneural, classical and mesenchymal subtypes [4,5], the scRNA-seq showed that all tumor samples consisted of heterogeneous mixtures with individual cells corresponding to different glioblastoma subtypes. Panoramic analysis of the chromosomal landscape identified chromosomal aberrations in each tumor cell, such as the gain of chromosome 7 and the loss of chromosome 10, the two most common genetic alterations in glioblastomas [82]. Single-cell transcriptomic analysis have revealed that IDHwt glioblastoma samples contain multiple cell states with distinct transcriptional programs and have provided inferential evidence for dynamic transitions; cell cycle-related genes were active in 1.4% to 21.9% of malignant cells. Application of the stemness signature revealed stemness gradients in all tumors, modestly anti-correlated to the cell cycle signature and consistent with the notion that NSCs divide at lower overall rates, as compared with IPCs. The stemness signature was stronger in individual cells from samples of proneural and classical subtypes. In contrast, cells of the neural subtype were more like oligodendrocytes. These findings suggested parallels between intratumoral cellular heterogeneity in glioblastomas and cellular diversity in the developing brain, with respective subsets of tumor cells resembling a stem cell and progenitor compartment, an astrocytic lineage, or an oligodendrocytic lineage. The analysis also revealed “hybrid” states in which a single cell scored highly for two subtypes, most commonly classical and proneural (progenitor states) or mesenchymal (differentiated states).

To further understand glioblastoma transcriptional and genetic heterogeneity, Neftel et al. addressed an integrative approach, combining scRNA-seq, analysis of bulk specimens and lineage tracing in glioblastoma models [83]. They found that malignant cells in glioblastomas may be grouped into four categories: neural progenitor-like (NPC-like), OPC-like, astrocyte-like (AC-like) and mesenchymal like (MES-like) states. While each glioblastoma sample contained cells in multiple states, the relative frequency of each state varied between tumors. Furthermore, by coupling scRNA-seq to uniquely barcoded single cells in vivo, Neftel et al. demonstrated the plasticity between states and the potential of a single mutated cell to generate all four states. This work provided a roadmap of the cellular programs of malignant cells in glioblastomas, as well as their plasticity and modulation by genetic drivers, but did not address the origin of the malignant cell types [83].

**Figure 4:**
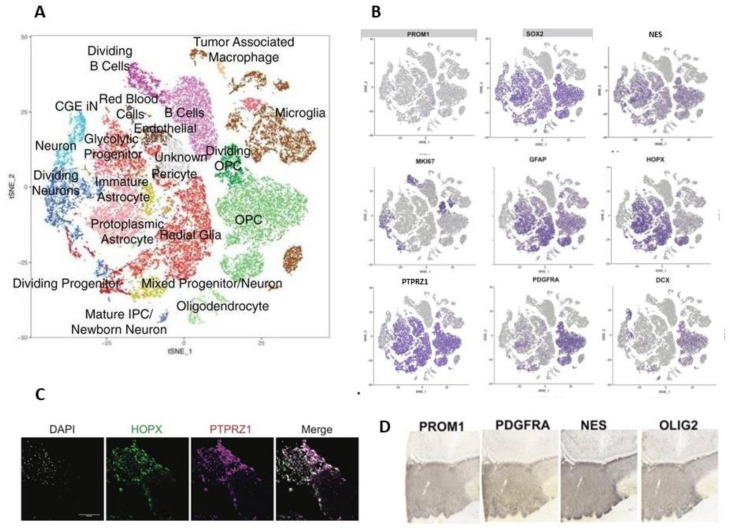
Single-cell RNA sequencing in glioblastoma. (**A**). Plot of cells from glioblastoma samples from 16 patients showing clusters colored by attributed cell types. (**B**). The same cluster representation as in (**A**), showing the cell-type/cluster-related expression of several genes expressed during neural development in stem and progenitor populations. (**C**). Immunofluorescence image of a glioblastoma sample showing co-expression of the oRG markers HOPX and PTPRZ1; DAPI nuclear staining (blue); scale bar: 10 μm. (**D**). Glioblastoma samples from a glioma repository, showing the expression of the proteins PROM1, PDGFRA, nestin (NES) and OLIG2. (Adapted from [84]).

Bhaduri et al. analyzed the glioblastoma samples by using scRNA-seq and the previously described transcriptional signatures of the developing human brain [73] and adult cortex [66]. Despite their heterogeneous composition, each tumour contained a distinctive combination of transcriptionally defined cancer cell types (Figure 4A) and a mitotic index of 20% or higher. By exploring the cell types associated with CNV, the authors found dividing RG-like cells, IP-like cells and OP-like cells, which were expected, but also neuronal-like cells which expressed *MKI67* (Figure 4B) [84]. 

Almost all identified cell types expressed at least one marker associated with stemness and previously known GSC markers. A number of previous studies have shown the potential for a variety of cell types to become GSCs, such as OPCs, astrocytes, and neuronal cell types [85,86,87,88]. However, the gene combinations previously associated with GSC stemness were expressed uniquely for each individual tumour. While several progenitor genes such as *SOX2* and *NES* were expressed broadly, *PROM1* (expressed as CD133), a marker that has been shown to be sufficient to give rise to ectopic tumours [7], was very sparsely expressed (Figure 4B). Thus, the cell types that make up glioblastomas can be found in various combinations across tumours, but the cocktail of stemness markers co-expressed within a GSC cell type is largely specific to every individual tumour. By further exploring a glioblastoma repository [89], Bhaduri et al. observed that individual tumours co-expressed a variety of GSC marker genes such as *PROM1* (CD133), *PDGFRA*, *NES* and *OLIG2* (Figure 4D), which also confirmed the expression found at the single cell transcriptomic level (Figure 4B). These results from orthogonal datasets support the hypothesis that diverse sets of GSCs can be found within a single tumour, characterized by heterogeneous marker gene combinations. The transcriptomic profiles of glioblastomas suggest that programs associated with stemness are broadly expressed, and that the activation of stemness programs indicated by these GSC marker genes can occur in almost any cell type within the tumour. Additionally, a distinct cell type within the glioblastoma atlas expressed oRG marker genes [84]. The oRG network was strongly recapitulated in glioblastoma, with the same hub genes such as *PTPRZ1* and *HOPX* [23,24] (Figure 4B), and with confirmed expression at the protein level (Figure 4C). This suggests that re-expression of the developmental oRG signature in GSCs is associated with a dynamic cell behaviour characteristic of prenatal oRG cells [84].

The oRG-like population in glioblastoma cells was further enriched using FACS PTPRZ1-positive selection and re-analyzed by scRNA-seq. Even though the PTPRZ1-positive sorted population was not homogenous, it was significantly enriched for RG-like cells compared to the PTPRZ1-negative population [90,91]. To functionally investigate the PTPRZ1-positive, negative and unsorted cell populations, they were labelled with a GFP-expressing adenovirus and transplanted into an in vitro model of human brain organoids. Two weeks after transplantation, the tumour cell populations were composed primarily of either neuronal or astrocytic cells. Both PTPRZ1-positive and negative cells expressed canonical GSC markers in each of these populations, consistent with the earlier observation that different glioblastoma cell types express stemness markers. The expression of GSC markers uniformly decreased after transplantation, while differentiated cell types within each population increased. Together, these results supported the PTPRZ1-positive oRG-like glioblastoma cells as being one of several GSC cell types and showed their invasive nature and involvement in tumor propagation [84].

**Figure 5:**
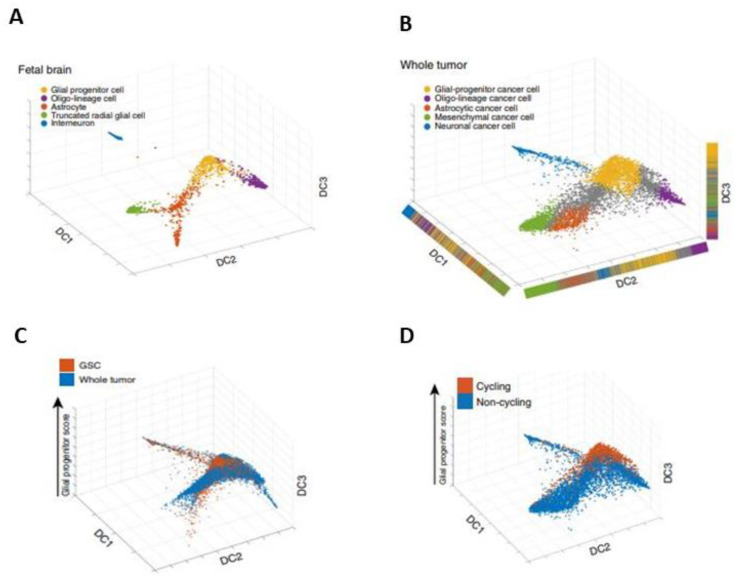
Developmental-related transcriptomic roadmaps in the human fetal brain and glioblastoma samples. Cell maps built with human prenatal telencephalic cells (**A**) and glioblastoma cells from whole-tumor samples (**B**–**D**) or enriched by CD133-positive selection; a population named glioma stem-like cells (GSC) in **C**. GPC cluster (orange, (**A**,**B**)), oligo-lineage cluster (violet, (**A**,**B**)), astrocyte cluster (coral, (**A**,**B**)), tRG cluster (green, (**A**,**B**)) and interneuron cluster (blue, (**A**,**B**)), but also a non-defined intermediate population (grey, (**B**); adapted from [74].

Couturier et al. made a step forward in defining the cells of origin and heterogeneity in glioblastoma by comparing the previously established lineage hierarchy of the developing human brain (Figure 5A) to the transcriptome at the single-cell level of both whole-tumor samples and CD133-positive selected samples (with the aim of increasing the proportion of GSCs), and after CNV selection. Plotted on the roadmap for human prenatal telencephalic cells (Figure 5A), the whole tumor samples mainly mapped onto a GPC cluster, an oligo-lineage cluster, an astrocyte cluster, a tRG cluster, and an interneuron cluster—but also onto a non-defined intermediate population (Figure 5B). In the population enriched by CD133-positive selection (named GSC in Figure 5C), most of the plotted cells expressed GPC genes, but some expressed neuronal and astrocytic genes. These data suggest that the GSC-enriched population is also heterogeneous but organizes into subpopulations resembling a developing brain [74].

Almost all cycling cancer cells had high glial progenitor scores (Figure 5D). These data also show that GPC-like cancer cells are the cell types with the highest rates of proliferation—more than the cancer cells undergoing lineage differentiation. This model reveals a GPC-centered organization in both the whole-tumor and the GSC-enriched population. Remarkably, the GPC signature was the only one robustly expressed in all patients. The identification of highly proliferative GP-like cells was in contrast to previous works [78,83], where such a population was not found. Protein marker panels, representative of each cancer cell type, and single-cell proteomic analysis, were additionally used to validate this result. Together, these analyses suggest that astrocytic, mesenchymal, oligodendrocytic, and neuronal-like glioblastoma cells are more differentiated than GP-like cells, and that they are one of the originators of the trilineage hierarchy in glioblastomas [74].

## 6. Towards Signaling-Specific Targeted Therapy in Gliomas

Significant obstacles hampering the development of effective cancer therapeutics include tumor heterogeneity and the persistence of GSCs that give rise to cancer recurrence. Most studies consider the GSC population to be uniform. However, recent single-cell transcriptomics studies have shown that GSCs display heterogeneity driven by a hierarchical developmental organization [74,81,84]. Some of these recent studies have evaluated chemoresistance and tumorigenicity in selected GSC populations in glioblastomas. In addition, identifying the signaling pathways that maintain tumor-initiating cell proliferation may provide therapeutic targets for inhibiting tumor growth. Identification of signaling pathway alterations between progenitor cancer cells and more differentiated cancer cells may yield meaningful new therapeutic targets.

The association of the genetic alterations in signaling pathway component genes such as *PDGFRα*, *EGFR* and *NF1* was explored in *IDH*wt glioblastomas, with each mutation being shown to favor a particular state [83]. However, these signaling pathway components are expressed broadly in different normal populations in the adult brain, making the targeted approach difficult. A more efficient approach should address signaling pathways related to more specific cell populations.

The proliferation and migration of the RG in the fetal brain occurs in response to different signals, parts of different signalling pathways, such as the FGF-MAPK, PI3K/PTEN/AKT, Hedgehog, N-cadherin/β-catenin, Notch, mTOR and Rho/Rho-kinase (ROCK) pathways [26,29]. Nowakowski et al. highlighted the gene enrichment at the single-cell level in oRG for *GLI2*, *NFAT2C,* and several regulators of the mTOR signalling pathway, as well as increased phosphorylation of the S6 ribosomal protein [73]. Bhaduri et al. explored the role of PTPRZ1 in oRG-like GSCs. PTPRZ1 and its ligand, PTN, have been previously identified as necessary for tumour invasion and viability and linked with the known effects of the Rho/Rho-kinase signalling pathway in glioblastoma [92,93,94]. The authors used genetic and pharmacologic approaches and found that *PTPRZ1* and *PTN* double knockdown significantly reduced the migration of the oRG selected from fetal human brains. In order to relate these findings to invasive behaviour in glioblastoma, an in vitro invasion assay used tumour samples treated with either control shRNAs, *PTPRZ1* shRNAs, or Rock inhibitor. Both *PTPRZ1* knockdown and Rock pathway inhibition significantly decreased the invasive behaviour of oRG-like cells in an in vitro model using human brain organoids, suggesting a selective way of further addressing single members of the GSC population [84].

Couturier et al. focused on GP-like cells as the originators of the cancer cell hierarchy in glioblastoma [74]. These rapidly cycling progenitor cancer cells were seen as a prime cell population to target and were tested both in vitro and in vivo, in xenograft models. In GSC culture conditions, where all GSCs retain the ability to divide, GP-like cells were found to be the most resistant to chemotherapy. Investigating the scRNA-seq transcriptomic data, Couturier et al. identified several pathways with a significant enrichment in the GPC cluster, as compared to the astro-mesenchymal groups. Hits with significant and strong correlations were found in pathways previously established as relevant to GSC self-renewal and tumorigenicity, such as the WNT pathway and the EZH2 and FOXM1 genes, but also in pathways of previously unknown significance in glioblastoma. Of these, the E2F4 pathway was the most significant, and was thus selected for testing. While E2F4 expression in glioblastoma tissue has been previously shown [95], Couturier et al. provided the first description of its role in the GPC malignant population. The E2F gene family regulates the cell cycle and is important for progenitor cell survival [96]. It has been shown that E2F4 inhibition causes senescence of gastric cancer cells [97]. The effect of the small molecule inhibitor HLM006474, which prevents E2F4 binding to DNA, was tested in vitro and in vivo in IDHwt glioblastoma cells. Following HLM006474 treatment, the proliferation and survival of the GPC population in vitro was reduced significantly as compared to the neuronal and astro-mesenchymal populations, supporting its specific effect on the GP-like cell population in glioblastomas. After orthotopically xenografting glioblastoma cells, a significant reduction in tumor growth and improved survival in the HLM006474-treated mice was observed. In addition, no synergism or antagonistic effect was found between the HLM006474 and classical chemotherapy. In addition, mice xenografted with GP-like cancer cells developed tumors faster and exhibited a shorter survival time than mice engrafted with OPC-like cancer cells [74].

While targeting the most rapidly cycling and functionally aggressive progenitor cancer cell population may be an effective treatment approach, given the plasticity that can occur in the GSC population, separate targeting of all cell types within a cancer may need to be addressed in future for each GSC subtype. The signalling pathway components waiting for further exploration in selected cell populations from glioma include the oRG-like and GPC-like cells. Several combined targeted therapies could address each tumor and cell type in a personalized approach.

## 7. Conclusions and Outlook

**Figure 6:**
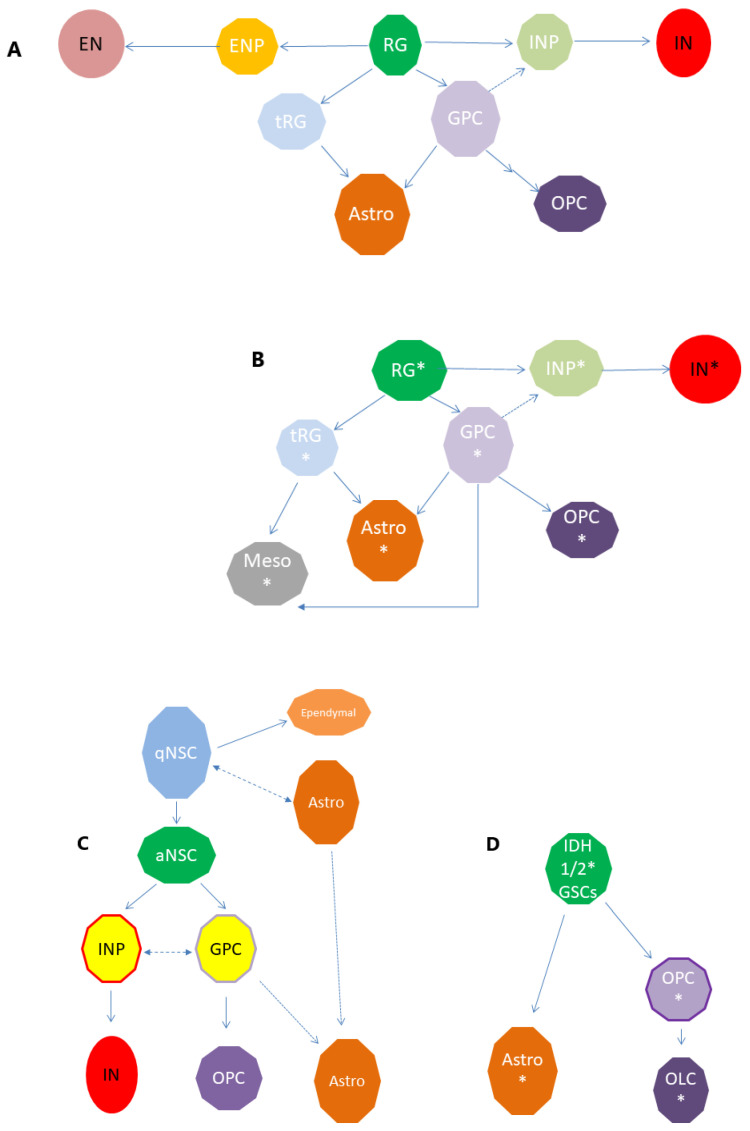
Towards a model of cell hierarchies in gliomas. Single-cell transcriptomics-based links proposed for the developing human telencephalon (**A**), adult human telencephalon (**C**), and the potential similarities with IDHwt glioblastomas (**B**) and IDH1/2 gliomas (**D**). The nodes in (**A**) represent the cell types from human fetal telencephalic samples corresponding to different types of radial glia (RG), excitatory neuron progenitors (ENPs), excitatory neurons (ENs), inhibitory neuron progenitors (INPs), inhibitory neurons (INs), truncated RG (tRG), astrocytes (Astros), glial progenitor cells (GPCs) and oligodendrocyte progenitor cells (OPCs; shown as clusters in Figure 2). Nodes in (**B**) marked with (*) represent cell types with IDHwt glioblastoma-related mutations. Mesenchymal-like cells (Mesos). The nodes in (**C**) represent the cell types in the adult subventricular zone (SZV) samples corresponding to different types of adult neural stem cells (NSCs), such as quiescent (qNSCs) and activated neural stem cells (aNSCs), inhibitory neuron progenitors (INPs), inhibitory neurons (INs), astrocytes (Astros), glial progenitor cells (GPCs) and oligodendrocyte progenitor cells (OPCs; shown as clusters in Figure 3). Nodes in (**D**) marked with (*) represent cell types with IDH1/2 glioma-related mutations. GSC: glioma stem-like cells, OLC: oligo-lineage cells.

Several transcriptomic-related studies have shown that the optimal classification of cell subtypes in a tissue or a tumour sample should imply a deeper knowledge of their origins and trajectories during development and maturation. The single cell transcriptomic approaches presented here provide convergent evidence for a robust description of cell type identity in both normal and pathological lineages, validating the neurodevelopment pathways as important players in tumour development. The developmental roadmaps generated from the transcriptomic studies still wait for additional work for validation, such as cell fate mapping in vitro and in vivo, which is necessary to uncover the exact position of these cells within the developmental hierarchy of the brain. However, the proposed models of cell hierarchies resemble a pattern that has also been shown in gliomas: GSCs generate daughter cells that subsequently differentiate into tumour bulk cells [93,98]. Using these tools, the cellular hierarchies proposed for the prenatal telencephalon can be linked with a diverse range of cerebral tumours, as we propose here for IDH-mutated glioma and IDHwt glioblastoma (Figure 6).

The single-cell profiles from IDH-mutated glioma and IDHwt glioblastoma appeared to be different, suggesting that different progenitor populations maintain their cell pool while producing additional tumour cell types. IDH-A and IDH-O share the same developmental hierarchy, consisting in each case of three subpopulations of malignant cells: two nonproliferating populations differentiated along the astrocytic and oligodendrocytic lineages, and a proliferative population of GSCs that resembles NSCs.

For the IDHwt glioblastoma, different GSC populations were detected, which exist at the intersection of neuronal, glial and mesenchymal lineages. These GSCs correspond to different classes of stem cells and progenitors found in human fetal telencephalon, including oRG and GPCs [74,84], which support the high degree of heterogeneity in this tumour type. Single-cell analysis suggests that progenitor cancer cells have the potential to differentiate into all the cancer cell lineages identified. GSC cell type-specific xenograft models show evidence of both neuronal and glial lineage commitment.

The transcriptomic analysis of Bhaduri et al. indicates that RG-like glioblastoma cells, along with other progenitor populations, might serve as tumour propagating cells in glioblastoma. ORG are stem cells of the developing human brain which give rise to a transit-amplifying progenitor cell population, and further to neurons and glia in a temporal- and lineage-dependent fashion [84].

The roadmap built in parallel with the fetal human telencephalon as a training set using an equal number of cancer cells from each of the analyzed *IDH*wt [74] revealed that glioblastoma develops along conserved neurodevelopmental gene programs and contains a rapidly dividing progenitor population, which corresponds in development to a newly defined glial progenitor type. These GPCs share markers with NSCs, but also to OPCs, and could be clearly clustered only in the CD133-positive selected samples. These data also show that tumour initiating cells or GSCs are the cancer cell type with the highest rates of proliferation—they were identified in glioblastoma and phenotyped only after enrichment for stem cell-like populations using CD133-positive selection, followed by scRNA-seq [62,63,99,100]. These results suggest a model in which developmental programs are reactivated in IDHwt glioblastoma cells by specific mutations (Figure 6A,B).

Another possibility is that different adult NSCs in the neurogenic and gliogenic niches are affected directly by these mutations, and that these cells share phenotypes with the population found during development. This is more likely to be a model for IDH1/2 gliomas, which may originate from adult stem cells (Figure 6C,D). However, as scRNA-seq of the adult SVZ niche cells was performed only for the adult rodent SVZ, further investigations of the human SVZ are expected to confirm this pathway. Again, additional work such as cell fate mapping in vitro and in vivo will also be necessary for the adult NSC, transit-amplifying, and migrating cell populations.

Taken together, transcriptomic analysis in normal brain development reconciles glioblastoma development, suggesting possible origins for the glioblastoma hierarchy, and helping to identify cancer stem-like cell-specific targets. However, the transcriptomic phenotypes of the GSC in IDHwt glioblastomas and in IDH-mutated gliomas are not the same. This suggests a different cell of origin for these pathologies than in adult IDHwt glioblastoma and may underlie the disparate natural histories and treatment responses between these cancer types.

A better understanding of the spectrum and dynamics of cellular states in several types of glioma is critical for establishing faithful models and advancing therapeutic strategies that address the complexity of this disease. Further combining the single-cell transcriptomic profiles of normal and tumour cells from the same brain region can provide the basis to better define the potential origins of neural cells initiating different types of primary cerebral tumours, and the design of therapies targeting GSC phenotypes—a potentially novel avenue in the treatment of these currently incurable malignancies.

Single-cell transcriptomic studies have started to rewrite the knowledge regarding the composition of the brain and its tumors, but also of cell hierarchies and cell-specific signaling. They belong to an ongoing major collaborative effort directed to generating a complete description of cell types in the human brain (the National Institutes of Health (NIH) BRAIN Initiative Network, braininitiative.nih.gov, accessed on 9 November 2021), in the human neocortex (Allen Institute for Brain Science, www.allenbrain.org, accessed on 9 November 2021), the whole human body (the Human Cell Atlas, www.humancellatlas.org, accessed on 9 November 2021) [62,63,99,100], and the pathological brain, such as the IVY glioblastoma repository [89].

As complementary approaches, several newly released methodologies, such as in situ sequencing of fixed tissue—called spatial transcriptomics [101,102,103,104]—offer important information needed to interpret the results obtained from single-cell transcriptomics. They enrich the picture by addressing each single cell in its tissue context. On this line, single-cell analyses and spatial transcriptomics are expected to be together applied to better characterize the cells that constitute the nervous system and its tumors [100].
